# A Novel Highly Durable Carbon/Silver/Silver Chloride Composite Electrode for High-Definition Transcranial Direct Current Stimulation

**DOI:** 10.3390/nano11081962

**Published:** 2021-07-30

**Authors:** Lingjun Li, Guangli Li, Yuliang Cao, Yvonne Yanwen Duan

**Affiliations:** 1 College of Chemistry and Molecular Sciences, Wuhan University, Wuhan 430072, China; lingjunli@whu.edu.cn; 2 Hunan Key Laboratory of Biomedical Nanomaterials and Devices, College of Life Sciences and Chemistry, Hunan University of Technology, Zhuzhou 412007, China; 3 Wuhan Greentek Pty. Ltd., Wuhan 430074, China

**Keywords:** HD-tDCS, carbon nanotube, Ag/AgCl, stimulation electrode, service life

## Abstract

High-definition transcranial direct current stimulation (HD-tDCS) is a promising non-invasive neuromodulation technique, which has been widely used in the clinical intervention and treatment of neurological or psychiatric disorders. Sintered Ag/AgCl electrode has become a preferred candidate for HD-tDCS, but its service life is very short, especially for long-term anodal stimulation. To address this issue, a novel highly durable conductive carbon/silver/silver chloride composite (C/Ag/AgCl) electrode was fabricated by a facile cold rolling method. The important parameters were systematically optimized, including the conductive enhancer, the particle size of Ag powder, the C:Ag:PTFE ratio, the saline concentration, and the active substance loading. The CNT/Ag/AgCl-721 electrode demonstrated excellent specific capacity and cycling performance. Both constant current anodal polarization and simulated tDCS measurement demonstrated that the service life of the CNT/Ag/AgCl-721 electrodes was 15-16 times of that of sintered Ag/AgCl electrodes. The much longer service life can be attributed to the formation of the three-dimensional interpenetrating conductive network with CNT doping, which can maintain a good conductivity and cycling performance even if excessive non-conductive AgCl is accumulated on the surface during long-term anodal stimulation. Considering their low cost, long service life, and good skin tolerance, the proposed CNT/Ag/AgCl electrodes have shown promising application prospects in HD-tDCS, especially for daily life scenarios.

## 1. Introduction

Transcranial direct current stimulation (tDCS) is a non-invasive neuromodulation technique that uses a constant low-intensity direct current (1~2 mA) to regulate the activity of cortical neurons [1,2,3,4]. In recent years, tDCS has been widely used in the clinical intervention and treatment of epilepsy [5,6,7,8], depression [9,10] stroke [11,12], and other neurological or psychiatric disorders [13,14,15,16,17,18]. Compared with other non-invasive brain stimulation techniques, tDCS has the advantages of high safety, good tolerance, convenient use, and less side effects [19,20,21,22,23]. The tDCS devices are often portable and can be integrated into existing wearable devices, so as to extend the application scenarios from traditional clinical laboratories to daily life. Many case studies have also confirmed the necessity of home tDCS treatment [24,25,26,27]. 

As an indispensable component of the tDCS device, tDCS electrodes (usually including anode and cathode) are mainly responsible for injecting the stimulation current into skin [17,28]. Two tDCS electrodes are placed on the specific scalp locations to form a loop to deliver a constant current into stimulated sites. In clinical practice, tDCS electrodes are often divided into two categories. The first category is conventional inert electrodes. The typical inert electrodes are stainless steel and conductive rubber electrodes [29], which are usually used with a saline-soaked sponge as the electrolyte [30,31]. Obviously, these inert electrodes cannot undergo any electrochemical reactions. During tDCS, the electrolysis of NaCl solution often occurs [32], which may cause a pH change of the skin tissue around the tDCS electrode, thus increasing the risk of skin irritation and injury. Due to the charge accumulation at the electrode/electrolyte interface, these inert electrodes cannot deliver high current density. Therefore, the area of inert electrodes has to be quite large (25~35 cm^2^), which severely sacrifices the spatial resolution of tDCS [33,34,35]. In general, the current density of inert electrodes is less than 0.08 mA cm^−2^.

In order to improve the spatial resolution, the second category (viz. sintered Ag/AgCl electrodes) that tolerate high current density is usually used. For example, small-sized sintered Ag/AgCl electrodes (less than about 2 cm^2^), together with conductive gels, are frequently used in HD-tDCS [36,37,38]. A representative 4 × 1 HD-tDCS electrode configuration consists of a single anode placed on the region of interest and four surrounding cathodes [31,35,39]. Such an electrode configuration decreases the contact area, and improves the tolerable stimulated current density and spatial resolution significantly [28]. The electrode can tolerate high current density up to 1 mA cm^−2^ roughly, which is more than 12 times that of inert electrodes (0.08 mA cm^−2^). Due to high spatial resolution and excellent tolerance to high current density, the sintered Ag/AgCl electrodes have become the preferred choice for HD-tDCS. Unlike inert electrodes, a fast and reversible electrochemical reaction occurs at the sintered Ag/AgCl electrode/electrolyte interface [40,41,42,43], allowing high current density pass through. Therefore, the interface impedance between electrode and electrolyte (i.e., saline, conductive gels) is very low [44]. In addition, the electrolysis of conductive gels hardly occurs, which effectively avoids the pH change of the skin tissue around the electrodes and reduces the risk of skin damage. 

However, the sintered Ag/AgCl electrodes often suffer from the issue of a short service life, especially for successive anodal HD-tDCS. Although the sintered Ag/AgCl electrodes were randomly used as an anode or a cathode in clinical practice, the non-conductive AgCl was still accumulated on the electrode surface after multiple anodal HD-tDCS [32,45], leading to an increase in electrode impedance and stimulation failure eventually. In addition, the non-uniform change in surface impedance may affect the stimulation electric field and the stimulation efficacy [46]. Langenbach et al. [46] found that repeated anodal HD-tDCS for eight times might render sintered Ag/AgCl electrodes unreliable due to the high impedance. Alternating use the electrodes as an anode or a cathode might be a potential solution to this problem. However, most clinical users tend to use the sintered Ag/AgCl electrodes repeatedly for economical and logistical reasons [46,47]. Moreover, the repeated use of sintered Ag/AgCl electrodes is more suitable for non-clinical scenarios because of their low cost and convenience [24,26]. For the purpose of repeated use, developing highly durable and good performance non-polarized tDCS electrodes is highly desirable. 

Herein, a highly durable conductive carbon/silver/silver chloride composite (C/Ag/AgCl) electrode was fabricated for HD-tDCS. The conductive carbon doped in Ag/AgCl can maintain good conductivity and specific capacity even if non-conductive AgCl is excessively accumulated on the electrode surface during long-term anodal stimulation. In addition, doping conductive carbon can also improve the redox reversibility, ensuring a better cycle performance. These above two effects of doping conductive carbon can prolong the service life of tDCS electrodes synergistically. The C/Ag/AgCl electrode was fabricated by a facile cold rolling method, with conductive carbon, Ag powder, and polytetrafluoroethylene (PTFE) as the conductive enhancer, active substance, and binder, respectively. The important parameters, including the conductive enhancer, the particle size of Ag powder, the C:Ag:PTFE ratio, the saline concentration, and the active substance loading, were systematically optimized. Thus, the carbon nanotube/silver/silver chloride (CNT/Ag/AgCl-721) electrode demonstrated satisfactory specific capacity and cycling performance. The service life of the CNT/Ag/AgCl electrode was investigated by constant current anodal polarization and simulated tDCS measurement. Moreover, the preliminary assessment of skin tolerance of the CNT/Ag/AgCl-721 electrode was also carried out. 

## 2. Materials and Method

### 2.1. Chemicals and Materials

Silver nitrate, sodium citrate, sodium borohydride, sodium chloride, isopropanol, and ethanol were analytical grade and purchased from Sinopharm Chemical Reagent Co. Ltd. (Shanghai, China). From Aladdin Reagent Inc. (Shanghai, China), 60 wt.% PTFE was purchased. Industrial-grade CNTs (outer diameter of 50 nm, inner diameter of 20~30 nm, and length of 10~30 μm) were supplied by Tanfeng Graphene Technology Co. Ltd. (Suzhou, China). Commercial 2 μm silver powders were purchased from the Beijing Nonferrous Metals Research Institute (Beijing, China). Ketjenblack (KB), Super P and KS-6 commercial conductive carbon black were supplied by the Wuhan Battery Factory (Wuhan, China). Sintered Ag/AgCl electrodes (outer diameter of 12 mm, inner diameter of 6 mm, and thickness of 1 mm) and GT10 conductive gels (~3.0 wt.% NaCl) were bought from Wuhan Greentek Pty. Ltd. (Wuhan, China).

### 2.2. Preparation of C/Ag/AgCl Electrodes

Silver powders were synthesized by the chemical reduction of silver nitrate with sodium borohydride as reductant. In brief, 0.1 mol of silver nitrate and 0.2 mol of sodium citrate were completely dissolved into 1 L of deionized water under stirring at a low temperature (0~5 °C). An amount of 1.0 mol of sodium borohydride was also dissolved into another 100 mL of deionized water under an ice water bath. Then, the as-obtained sodium borohydride solution was added dropwise to the silver nitrate solution, and a black precipitate was formed immediately. Afterwards, the black precipitate was suction filtered, washed and dried to obtain silver powder. Different sizes of silver powders (0.1 and 0.5 μm) were prepared by controlling the dropping rate of sodium borohydride. 

The C/Ag/AgCl electrodes were fabricated by a cold rolling method, with Ag powder, conductive carbon, and PTFE as the active substance, conductive enhancer and binder, respectively. Firstly, a suitable amount of Ag powder, conductive carbon and PTFE was mixed and ground uniformly. Then, a suitable volume of isopropanol (the mass ratio of isopropanol:Ag powder plus conductive enhancer = 2:1) was added into the above solid mixture and stirred to form thick paste. Then, the as-obtained thick pastes were rolled to a thick film by a YLJ-15T roll squeezer machine (Jingke Materials Pty. Ltd., Hefei, China). The as-obtained film was dried in a vacuum oven at 60 °C overnight, and then cut into many discs with the required size (diameter of 12 mm, thickness of 0.5 mm). The discs were pressed on the titanium mesh, and silver wires were inserted into the electrode discal for subsequent signal transmission. One side of the electrode was encapsulated by epoxy resin. It was noted that the initial active substance of pure Ag was converted into Ag/AgCl when in contact with aqueous electrolyte solution (such as saline). In addition, Ag/AgCl was also gradually formed on the electrode surface during charging (or anodal stimulations). Therefore, the electrode was nominated as the carbon/silver/silver chloride composite (C/Ag/AgCl) electrode. 

**Figure 1:**
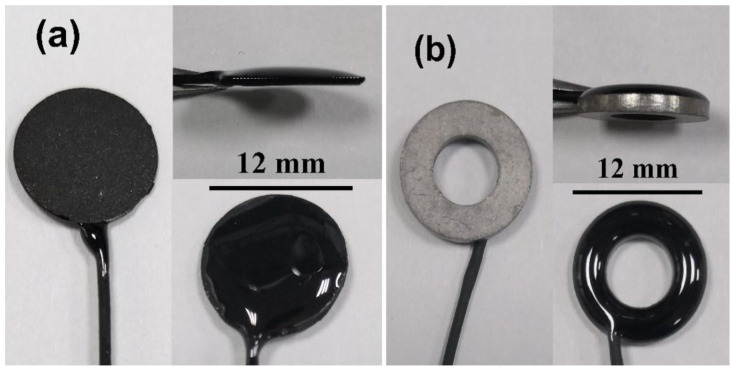
Photos of the (**a**) C/Ag/AgCl and (**b**) sintered Ag/AgCl electrodes.

The contact area of C/Ag/AgCl electrodes is 1.32 ± 0.21 cm^2^. Sintered Ag/AgCl electrodes were also used for comparison, and their contact areas are 1.41 ± 0.08 cm^2^. The photos of the C/Ag/AgCl and sintered Ag/AgCl electrodes are shown in Figure 1.

### 2.3. Optimization of the Preparation of C/Ag/AgCl

To pursue high durability and good performance, the conductive enhancer, particle size of Ag powder, the component mass ratio (Ag:C:PTFE), the saline concentration and the active substance loading were optimized systematically. In this section, the important parameters for fabricating the C/Ag/AgCl electrodes are summarized in Table 1. 

### 2.4. Material Characterization

The morphologies and crystalline phases of the as-obtained Ag powders and CNT/Ag/AgCl-721 were investigated by scanning electron microscopy (SEM) and powder X-ray diffraction (XRD). The SEM images were collected from a Merlin Compact scanning electron microscope (Carl ZEISS, Jena, Germany). XRD measurements were carried out by an XRD-6000 diffractometer (Shimadzu, Tokyo, Japan) operating at 30 kV and 30 mA with Cu Kα radiation (λ = 1.54056° A). Scans were typically carried out from 10 to 90° with a scan rate of 5° min^−1^. 

### 2.5. Electrochemical Measurements

Three-electrode configuration was used in all electrochemical experiments unless otherwise stated, consisting of a C/Ag/AgCl or sintered Ag/AgCl electrode, a carbon electrode, and a saturated Ag/AgCl electrode as the working electrode, the counter electrode and the reference electrode, respectively. The electrolyte was 3.0 wt.% saline unless otherwise stated. The constant current charging was performed by a Neware tester (Shenzhen Newell company, Shenzhen, China), with a charging current of 2 mA and cut-off voltage of 0.5 V. The plots of electrode potential versus time were recorded. The specific capacity can be estimated by dividing the product of charging current and time by the mass of active substance.

### 2.6. Simulated tDCS Measurement 

During simulated tDCS measurement, an agar block was used as a skin phantom since the electrical properties are similar to real skin. In addition, 0.9% NaCl solution can simulate the subcutaneous tissue well [40]. The agar skin phantom was prepared as follows. Firstly, 1.0 g of agar powder was added into 50 mL of heated 0.9 wt.% NaCl solution, and was stirred until the agar was completely dissolved. Then, the mixture was cooled to room temperature to form an agar block [40]. A two-electrode configuration was used for simulated tDCS experiments, consisting of two CNT/Ag/AgCl-721 electrodes as the working and counter electrodes, respectively. These two electrodes were placed on the surface of the agar skin phantom, at the center-to-center distance of 5 cm, approximately. An amount of 1.5 mL of GT10 conductive gels were injected between the electrode and agar block. The constant anodal current of 2 mA was applied to the two electrodes by an Autolab PGSTAT128N potentiostat (Metrohm Autolab BV, Utrecht, the Netherlands), and the measurements were automatically terminated when the output voltage reached 10 V. 

### 2.7. Preliminary Investigation of Skin Tolerance

Ten heathy volunteers (four females and six males, age between 21 and 35 years) were enrolled for the preliminary investigation of skin tolerance. The experimental protocol was approved by the Research Ethics Committee of Wuhan University, China, and written informed consent was obtained from all volunteers. Two CNT/Ag/AgCl-721 electrodes were placed on the right forearm of volunteers by an elastic cloth strip, with the center-to-center distance of 5 cm. GT10 conductive gels were applied between the electrode and skin. The HD-tDCS was applied to the skin for 30 s using a commercial stimulator (tDCS Mini CT, Soterix Medical Inc., New York, NY, USA) [48,49]. We asked the volunteers whether or not—in their opinion—they felt obvious irritation during tDCS with the CNT/Ag/AgCl-721 electrodes and we also collected their other comments regarding the skin sensations [50,51]. After stimulation, the skin under the tDCS electrodes was carefully examined. 

## 3. Results and Discussions

### 3.1. Physical Characterization of Ag Powders

**Figure 2:**
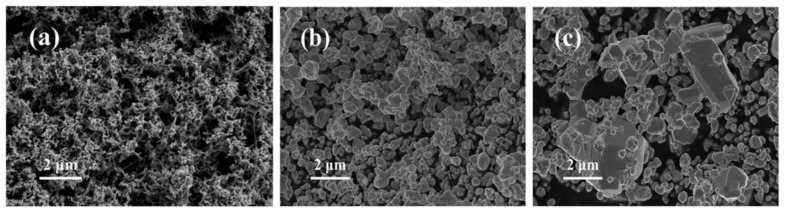
SEM images of 100 nm (**a**), 500 nm (**b**) and commercial 2 μm (**c**) Ag powders.

Nano-Ag powders with different particle sizes were prepared by controlling the dropping rate of sodium borohydride into a silver nitrate solution. Figure 2 shows the SEM images of nano-Ag and commercial Ag powders. As seen from Figure 2a,b, the flake-like Ag nanoparticles are uniformly dispersed with the average sizes of 100 nm and 500 nm, respectively. In contrast, the commercial Ag powders exhibit non-uniform and irregular shapes, composed of a large number of nano-silver flakes and a small number of micron-sized silver flakes (Figure 2c).

**Figure 3:**
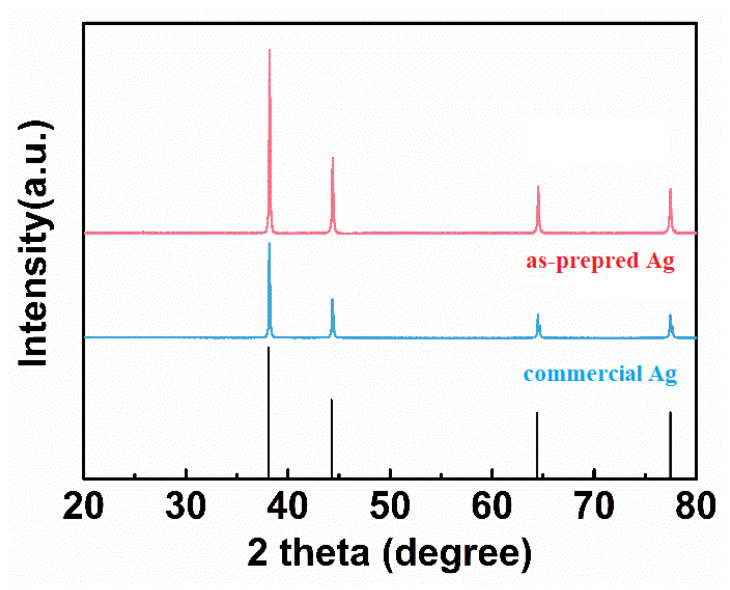
XRD patterns of the as-prepared and commercial Ag powders.

Figure 3 shows the XRD patterns of the as-prepared and commercial Ag powders. The apparent diffraction peaks at 38.1°, 44.3°, 64.4°, 77.4°, and 81.5° are observed in both the as-prepared and commercial Ag powders, which can be well assigned to (111), (200), (220), (311) and (222) facets of cubic Ag (JCPDS No.87-0597). Moreover, there is no obvious impurity peak, indicating that high-purity silver powders were synthesized successfully.

### 3.2. Optimization of Preparation of C/Ag/AgCl Electrodes

Anodal/cathodal tDCS are very similar to the constant current charging/discharging processes. Therefore, the durations of charging/discharging represent the service life of tDCS electrodes. For HD-tDCS applications, the stimulation current and the mass of active substance is constant; therefore, the specific capacity directly reflects the service life. In this section, the specific capacity and cycling performance under different conditions was investigated for optimizing the preparation of C/Ag/AgCl electrodes.

#### 3.2.1. Optimization of Conductive Enhancer

**Figure 4:**
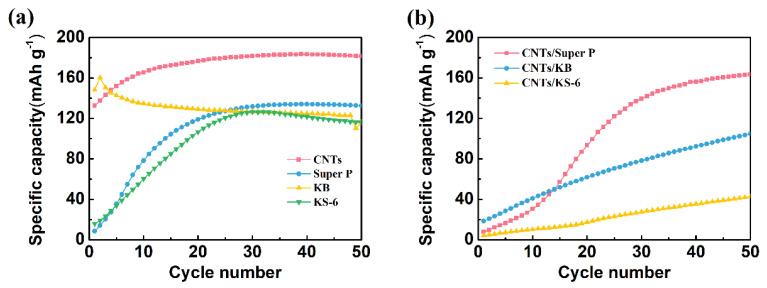
Charging cycling performance of the C/Ag/AgCl electrodes with four various conductive carbon materials (**a**) and three mixed CNTs with commercial carbon black (**b**) as conductive enhancer.

Four types of conductive carbon materials commonly used in batteries (including KS-6, Super P, KB and CNTs) were doped into the Ag powders. Then, the specific capacity of these four types of electrodes was compared. As shown in Figure 4a, the KB/Ag/AgCl electrode displays the highest specific capacity (160 mAh g^−1^) at the first cycle. However, its specific capacity degrades quickly, and drops down to 123 mAh g^−1^ after 50 cycles of charging. At the first cycle, the specific capacity of the CNT/Ag/AgCl electrode is 133 mAh g^−1^. To our surprise, its specific capacity gradually increases with the increase in the number of cycles, and achieves plateau (183 mAh g^−1^) at about 25 cycles, probably due to a rapid electrode activation process. In contrast, the specific capacity of both KS-6/Ag/AgCl and Super P/Ag/AgCl is very low. It must take a longer time to activate the electrode and maintain relatively high and stable specific capacity (about 130 mAh g^−1^). Obviously, these two tDCS electrodes are not suitable for practical applications. For the durable HD-tDCS applications, CNTs were preliminary selected as the conductive enhancers.

For further optimization of conductive carbon materials, the specific capacity of the mixed CNTs with commercial carbon black (1:1) was also investigated. Clearly seen from Figure 4b, the initial specific capacity of these three mixed CNT/Ag/AgCl is less than 20 mAh g^−1^. Only the CNT/Super P/Ag/AgCl electrode achieves 160 mAh g^−1^ after 50 cycles, suggesting that a long activation process is required to achieve a high and stable specific capacity. However, the specific capacity of the other two mixed CNT/Ag/AgCl electrodes is lower than 100 mAh g^−1^ even after 50 cycles. Moreover, the first charge specific capacity and cycling performance of the mixed CNT/Ag/AgCl electrodes is worse than the parent CNT/Ag/AgCl. Hence, CNTs were selected as the conductive enhancers in the following experiments.

#### 3.2.2. Optimization of Particle Size of Ag Powders

**Figure 5:**
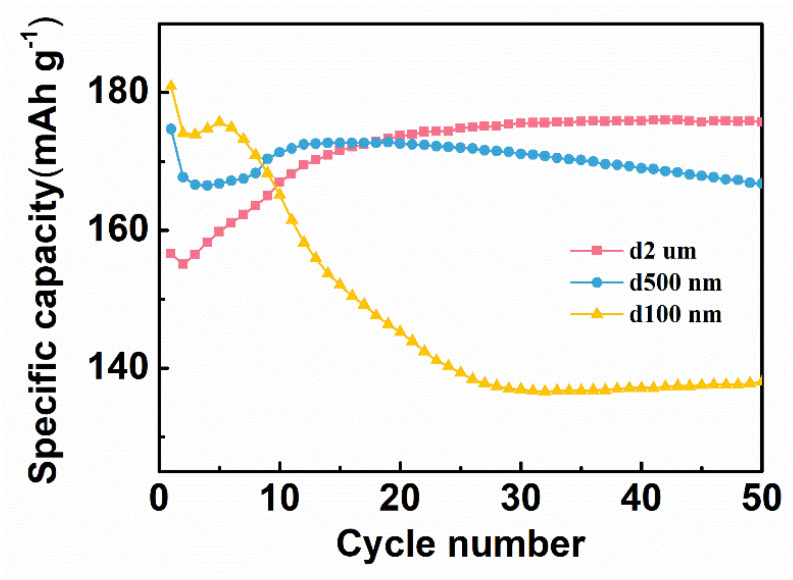
Specific capacity of three C/Ag/AgCl electrodes with different sizes of Ag powders.

The particle size of the Ag powder has a great influence on the specific capacity and cyclic reversibility. Therefore, the specific capacity of three C/Ag/AgCl tDCS electrodes with different sizes of Ag powders as electrode active materials was also investigated. As shown in Figure 5, the CNT/Ag/AgCl–0.1 μm has the highest specific capacity (181 mAh g^−1^) at the first cycle of charging, probably because of the smaller size of Ag powders, the better contact with electrolytes and more accessible active sites. However, its specific capacity degrades rapidly, and decreases to 138 mAh g^−1^ after 50 cycles. The CNT/Ag/AgCl–0.5 μm has a little lower specific capacity (175 mAh g^−1^) at the first cycle. However, its specific capacity decreases as the number of cycles increases and drops down to 167 mAh g^−1^ after 50 cycles. In contrast, the specific capacity of the CNT/Ag/AgCl–2.0 μm dramatically increases with the increase in the number of cycles, and achieves plateau (176 mAh g^−1^) at 20 cycles. The active substances in the CNT/Ag/AgCl–2.0 μm are hierarchical nano- and micro-size Ag flakes. Therefore, fewer active sites present in the commercial Ag powders result in lower specific capacity in the initial stage. As the number of cycles increases, the large Ag particles are gradually pulverized into small sizes to achieve electrode activation. For durable HD-tDCS, the high and stable specific capacity is very essential. Therefore, the commercial Ag powders were chosen as the active substance.

#### 3.2.3. Optimization of C:Ag:PTFE Ratio

**Figure 6:**
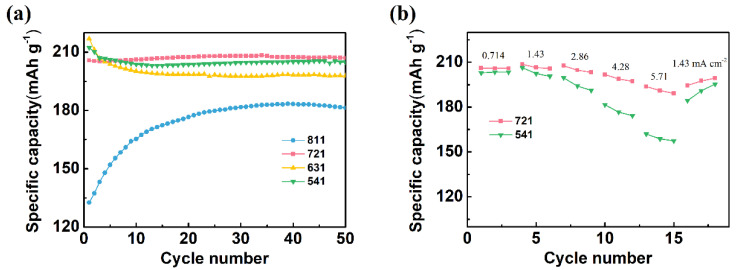
(**a**) Specific capacity of the CNT/Ag/AgCl electrodes fabricated with various CNT:Ag:PTFE mass ratios; (**b**) rate performance of the CNT/Ag/AgCl-721 and the CNT/Ag/AgCl-541 electrodes (*n* = 3).

The electrochemical performance of the CNT/Ag/AgCl electrode heavily depends upon the component mass ratio. Therefore, the effect of component mass ratio on the specific capacity and cycling performance was also investigated. Herein, the CNT/Ag/AgCl electrodes with the Ag:CNT:PTFE ratio of 8:1:1, 7:2:1, 6:3:1, and 5:4:1 were nominated as the CNT/Ag/AgCl-811, CNT/Ag/AgCl-721, CNT/Ag/AgCl-631, and CNT/Ag/AgCl-541, respectively. At the first cycle, the charging specific capacity of the CNT/Ag/AgCl electrodes tends to increase as the CNT ratio increases (Figure 6a). In addition, the charging specific capacity of CNT/Ag/AgCl-811 at the first cycle is much lower than the other three electrodes (above 200 mAh g^−1^). More CNTs in tDCS electrodes can form compact three-dimensional interconnected conductive networks, which make silver powder disperse uniformly, and thus effectively improve the contact area between active substance and electrolyte. However, excessive CNTs in tDCS electrode will decrease the total capacity, probably due to the negligible contribution of CNTs to the reversible capacity.

The specific capacity of the CNT/Ag/AgCl-721 and the CNT/Ag/AgCl-541 electrodes is very close at all cycles. For further optimization, the rate performance of these two tDCS electrodes were also investigated (Figure 6b). When charging at 0.714 mA cm^−1^, the specific capacity of these two tDCS electrodes almost resemble each other (~206 mAh g^−1^). However, the specific capacity of the CNT/Ag/AgCl-541 electrode dramatically decreases as the current density increases gradually. The CNT/Ag/AgCl-721 electrode still retains a stable specific capacity (191 mAh g^−1^) and even current density up to 5.71 mA cm^−2^. In addition, upon its return to 1.43 mA cm^−2^, its specific capacity recovers to the initial value (206 mAh g^−1^). Therefore, the optimal CNT:Ag:PTFE ratio was recommended as 7:2:1.

#### 3.2.4. Optimization of Saline Concentration

**Figure 7:**
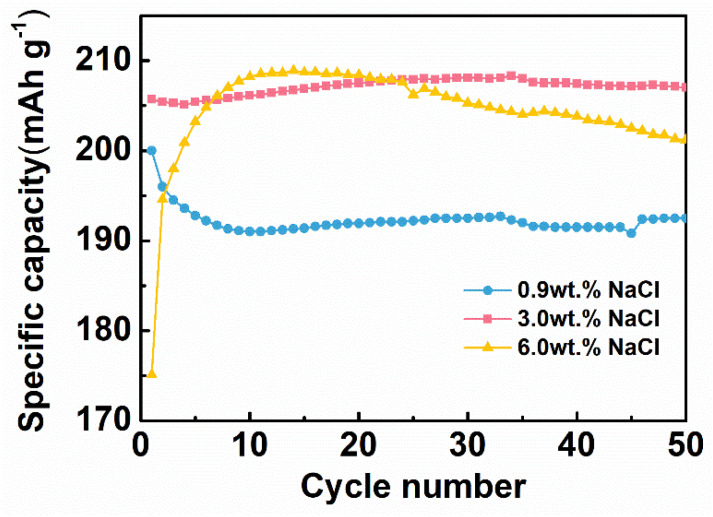
Specific capacity of the CNT/Ag/AgCl-721 electrodes in 0.9 wt.%, 3.0 wt.% 6.0 wt.% at different cycles (*n* = 3).

Cl^−^ is involved in the electrochemical reaction during HD-tDCS, so the saline concentration directly affects the specific capacity of the CNT/Ag/AgCl-721 electrode. The influence of saline concentration on the specific capacity was also explored. As shown in Figure 7, the CNT/Ag/AgCl-721 electrode in 3.0 wt.% saline outperforms the one in 0.9% and 6.0% saline in terms of both the charging specific capacity at the first cycle and specific capacity retention. The specific capacity of the CNT/Ag/AgCl-721 electrode in 0.9% saline is very close to the one in 3.0% at the first cycle. However, the specific capacity of the CNT/Ag/AgCl-721 electrode degrades gradually as the number of cycles increases to 10, probably because a low concentration of saline results in slow electrochemical kinetics on the electrode surface. In 6.0% saline, the specific capacity at the first cycle is relatively low. Then, the specific capacity suddenly increases and reaches the maximum values when the number of cycles increases to 10, mainly due to the electrode activation process. Afterwards, the specific capacity declines gradually, which may be because the high concentration of saline accelerates the electrochemical reaction, thereby yielding a dense and thick AgCl film on the electrode surface and thus leading to a capacity attenuation. Therefore, 3.0 wt.% saline was selected as the electrolyte in the following measurements.

Cl^−^ is involved in the electrochemical reaction during HD-tDCS, so the saline concentration directly affects the specific capacity of the CNT/Ag/AgCl-721 electrode. The influence of saline concentration on the specific capacity was also explored. As shown in Figure 7, the CNT/Ag/AgCl-721 electrode in 3.0 wt.% saline outperforms the one in 0.9% and 6.0% saline in terms of both the charging specific capacity at the first cycle and specific capacity retention. The specific capacity of the CNT/Ag/AgCl-721 electrode in 0.9% saline is very close to the one in 3.0% at the first cycle. However, the specific capacity of the CNT/Ag/AgCl-721 electrode degrades gradually as the number of cycles increases to 10, probably because a low concentration of saline results in slow electrochemical kinetics on the electrode surface. In 6.0% saline, the specific capacity at the first cycle is relatively low. Then, the specific capacity suddenly increases and reaches the maximum values when the number of cycles increases to 10, mainly due to the electrode activation process. Afterwards, the specific capacity declines gradually, which may be because the high concentration of saline accelerates the electrochemical reaction, thereby yielding a dense and thick AgCl film on the electrode surface and thus leading to a capacity attenuation. Therefore, 3.0 wt.% saline was selected as the electrolyte in the following measurements.

#### 3.2.5. Optimization of Active Substance Loading

**Figure 8:**
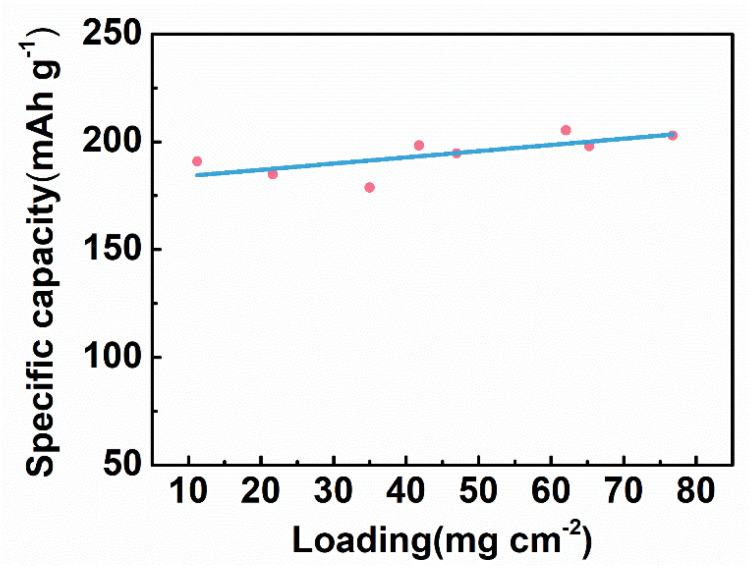
Specific capacity of the CNT/Ag/AgCl-721 electrodes with various active substance loading.

In general, the increase in the active substance loading (the active substance mass per unit area) leads to the decrease in the specific capacity. As the active substance loading increases, the electrode becomes thicker, which hinders the electrolyte penetration and ion migration, resulting in the decrease in the electrode capacity. For electrical stimulation, a higher total capacity of the tDCS electrode ensures a longer service life at the constant current mode. When increasing the active substance loading, the specific capacity can remain unchanged, and thus the effective stimulation time can be prolonged. Therefore, it is well worth optimizing the active substance loading. As plotted in Figure 8, the specific capacity of the CNT/Ag/AgCl-721 electrode is almost unchanged with the active substance loading ranging from 10 to 80 mg cm^−2^. Therefore, the service life of the CNT/Ag/AgCl-721 electrode can be extended by increasing the active substance loading in this range.

### 3.3. Constant Current Anodic Polarization

In general, tDCS electrodes used as anode and cathode alternately can prolong their service life effectively. However, tDCS electrodes are often used randomly as anode and cathode in clinical practice. Therefore, an electrode may be continuously subjected to anodal or cathodal direct current stimulation, which leads to over-oxidation and over-reduction. After injecting anodal current stimulation continuously, the impedance of sintered Ag/AgCl electrodes increases significantly, which is due to the formation of excessive non-conductive Ag/AgCl on the electrode surface by over-oxidation. The required output voltage raises greatly accordingly. Once the required output voltage exceeds the rated output voltage of tDCS stimulators, the anodal current cannot be effectively injected into human skin [46]. Therefore, the constant current anodal polarization of the CNT/Ag/AgCl electrode was also investigated. 

**Figure 9:**
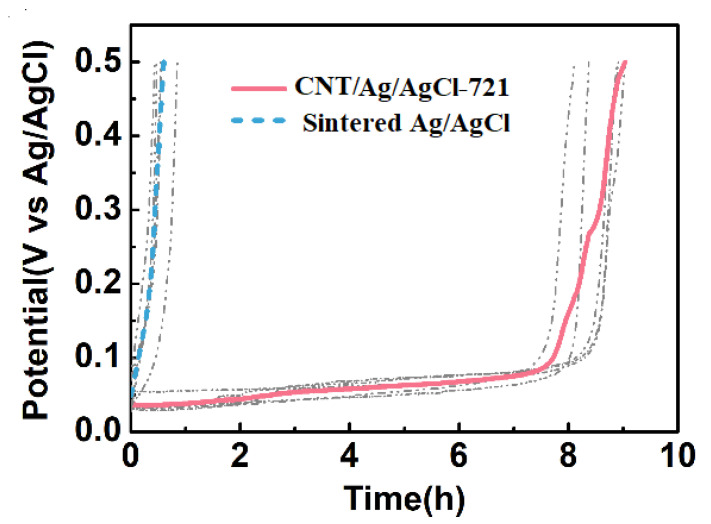
During the first cycle of charging, the electrode potential variation of the CNT/Ag/AgCl and the sintered Ag/AgCl electrodes with time.

The current density is often set as 1.4 mA cm^−2^ (the current is 2 mA) for most HD-tDCS studies [52,53,54]. Therefore, the constant current anodal polarization tests were performed at 1.43 mA cm^−2^, and the plots of electrode potential versus time were also recorded during the first cycle of charging (Figure 9). The time when the electrode potential reaches the cut-off voltage (0.5 V) is regarded as the service life of the electrode. At the first cycle, the specific capacity of the CNT/Ag/AgCl-721 electrode (192.3 mAh g^−1^) is much higher than that of the sintered Ag/AgCl electrode (4.156 mAh g^−1^). After introducing CNT into Ag/AgCl, the service life of the tDCS electrode extends from 0.58 to 8.5 h. The theoretical specific capacity of Ag is 248 mAh g^−1^, so the utilization factors of the electrode active substance in the CNT/Ag/AgCl-721 electrode and the sintered Ag/AgCl electrode are estimated as 77.54% and 1.676%, respectively. Owing to its high utilization factor of the electrode active substance, the cost of the electrode also reduces significantly.

**Figure 10:**
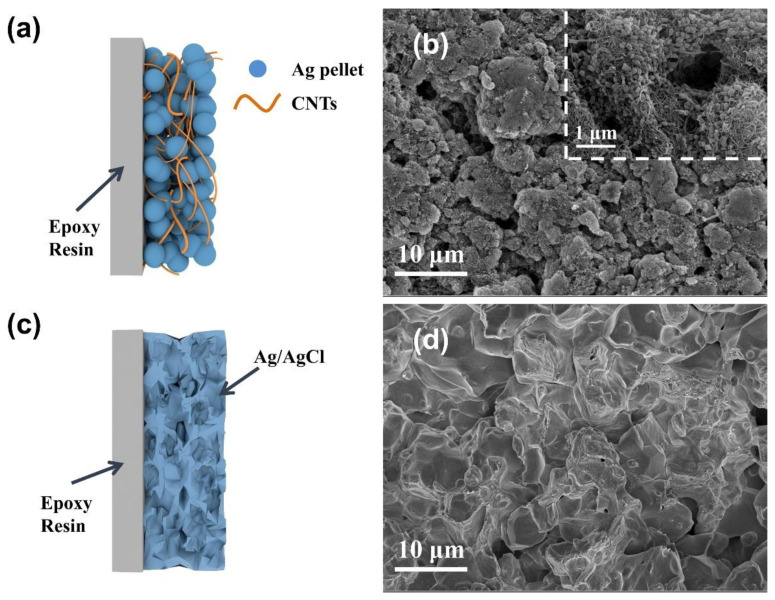
Illustrative diagram of the (**a**) CNT/Ag/AgCl-721 and (**b**) sintered Ag/AgCl electrodes; SEM images of the (**c**) CNT/Ag/AgCl-721 and (**d**) sintered Ag/AgCl electrodes.

The much higher specific capacity and longer service life can be attributed to the formation of the three-dimensional interpenetrating conductive network with CNT doping (Figure 10a,b), which promotes electron and ion transport and increases the contact of active sites with the electrolyte. The sintered Ag/AgCl electrodes displays a microporous structure due to the sintering at high temperature (Figure 10c,d).

**Figure 11:**
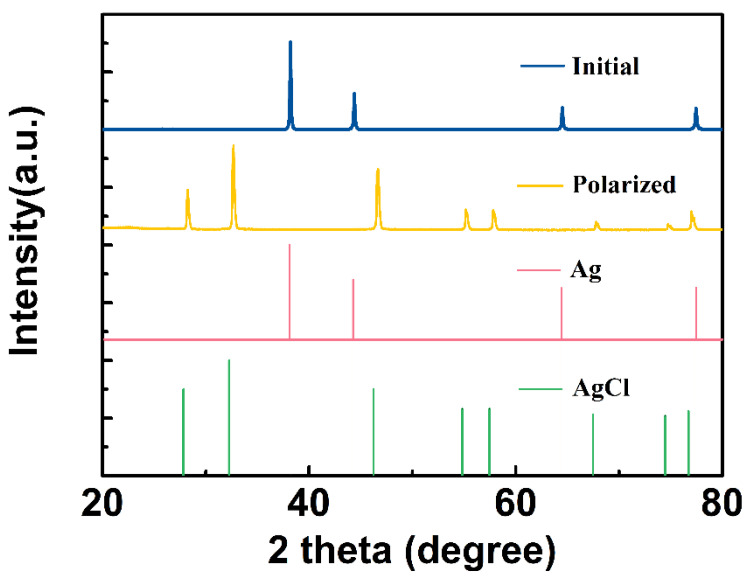
XRD patterns of the initial and anodal polarized CNT/Ag/AgCl electrodes.

During constant current anodal polarization, the chemical composition of the CNT/Ag/AgCl electrode was studied by X-ray diffraction (XRD). The XRD patterns of the initial and polarized CNT/Ag/AgCl-721 electrodes are shown in Figure 11. The characteristic diffraction peaks of the initial CNT/Ag/AgCl electrode are highly consistent with the standard powder diffraction card of JCPDS silver file (JCPDS No. 87-0597). After anodal polarization, all the characteristic diffraction peaks are consistent with the standard diffraction peaks of AgCl (JCPDS No. 31-1238), indicating the Ag was converted to AgCl.

### 3.4. Simulated tDCS Measurement

**Figure 12:**
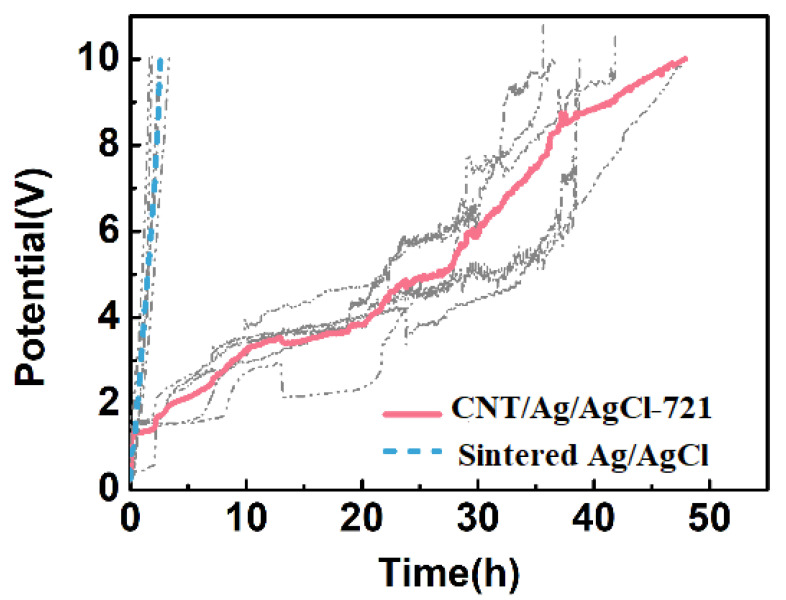
Electrode potential variation of the CNT/Ag/AgCl-721 and the sintered Ag/AgCl electrodes with stimulation time.

When the voltage exceeds the cut-off voltage (10 V), the electrode/skin impedance is higher than 5.0 kΩ, severely hindering the anodal or cathodal current injecting into the skin. Therefore, the voltage beyond 10 V can be used as an important index for electrode failure. The simulated tDCS measurements were performed on an agar skin phantom, and the results are shown in Figure 12. At the charging current density of 1.43 mA cm^−2^, the average working time of the CNT/Ag/AgCl-721 electrode is 48 h (*n* = 5), which is 16-fold of the one of the sintered Ag/AgCl electrodes (3 h, *n* = 5). With the continuous injection of an anodal current into the skin phantom, the non-conductive AgCl are gradually accumulated on the surface of the sintered Ag/AgCl electrode, which significantly increases the total impedance or voltage of the electrode and eventually leads to the failure of the electrode. When doping CNTs into the electrode, a good three-dimensional interconnected conductive network is formed, which can effectively hinder the rapid increase in impedance, and thus a higher charge is allowed to pass through the electrode. In practical applications, the tDCS electrodes are randomly used as anode and cathode. In addition, the time for a single application of tDCS is often less than 0.5 h. Therefore, the working times obtained from simulated tDCS measurements are not the actual working times during tDCS. Nevertheless, the simulated tDCS measurements demonstrate that the proposed CNT/Ag/AgCl-721 electrodes are more durable than the conventional Ag/AgCl electrodes.

### 3.5. Preliminary Evaluation of Skin Tolerance

The skin tolerance was preliminarily evaluated through a questionnaire survey. In general, previous studies have shown that skin irritation is primarily reported during the initial and end periods of tDCS, which corresponds to the stimulation current increase and decrease, respectively [48,49]. Therefore, three subjects were asked to report the skin sensations for the initial and end periods of tDCS. None of the subjects felt obvious irritation at the beginning and end of tDCS, suggesting the CNT/Ag/AgCl-721 electrode has good skin tolerance. Moreover, the skin beneath the tDCS electrodes was carefully examined after applying tDCS, and no redness or allergy was observed. These results suggest that the CNT/Ag/AgCl-721 electrode is very safe.

## 4. Conclusions

In this study, a novel highly durable conductive carbon/silver/silver chloride composite (C/Ag/AgCl) electrode was developed for HD-tDCS. The C/Ag/AgCl electrode was fabricated by a facile cold rolling method. The important factors affecting the specific capacity, including the conductive enhancer, the particle size of Ag powder, the C:Ag:PTFE ratio, the saline concentration, and the active substance loading were optimized systematically. The CNT/Ag/AgCl-721 electrode showed excellent specific capacity and cycling performance. At the first cycle of charging at 1.43 mA cm^−2^, the specific capacity of the CNT/Ag/AgCl-721 electrode (192.3 mAh g^−1^) is much higher than that of the sintered Ag/AgCl electrode (4.156 mAh g^−1^). The service life of the CNT/Ag/AgCl electrode extended from 0.58 to 8.5 h. The simulated tDCS experiments demonstrated that the service life of the CNT/Ag/AgCl-721 electrodes is 16 times that of the sintered Ag/AgCl electrodes. The much longer service life of the CNT/Ag/AgCl-721 electrodes can be attributed to the formation of the three-dimensional interpenetrating conductive network with CNT doping, which can maintain good conductivity and cycling performance even if excessive non-conductive AgCl is accumulated on the surface by long-term anodal stimulation. The preliminary evaluation of skin tolerance indicated that the proposed CNT/Ag/AgCl electrode is very safe. In summary, the proposed CNT/Ag/AgCl electrodes are very suitable for HD-tDCS, especially for daily life scenarios.

## Figures and Tables

**Table 1:** Parameters for the optimization of fabrication of the C/Ag/AgCl electrodes.

Optimized Parameters	Conductive Enhancer	Particle Size of Ag	Ag:C:PTFE Ratio	Saline Concentration	Active Substance Loading
Conductive enhancer	CNTs, KB, Super P, KS-6	Commercial 2.0 μm	8:1:1	3.0 wt.%	39 mg cm^−2^
Particle size of Ag	CNTs	0.1, 0.5 and commercial 2.0 μm	8:1:1	3.0 wt.%	39 mg cm^−2^
Ag:C:PTFE ratio	CNTs	Commercial 2.0 μm	8:1:1,7:2:1, 6:3:1, 5:4:1	3.0 wt.%	39 mg cm^−2^
Saline concentration	CNTs	Commercial 2.0 μm	7:2:1	0.9, 3.0 and 6.0 wt.%	39 mg cm^−2^
Active substance loading	CNTs	Commercial 2.0 μm	7:2:1	3.0 wt.%	10~80 mg cm^−2^

## Data Availability

All data are contained within the article.
